# Badges to Acknowledge Open Practices: A Simple, Low-Cost, Effective Method for Increasing Transparency

**DOI:** 10.1371/journal.pbio.1002456

**Published:** 2016-05-12

**Authors:** Mallory C. Kidwell, Ljiljana B. Lazarević, Erica Baranski, Tom E. Hardwicke, Sarah Piechowski, Lina-Sophia Falkenberg, Curtis Kennett, Agnieszka Slowik, Carina Sonnleitner, Chelsey Hess-Holden, Timothy M. Errington, Susann Fiedler, Brian A. Nosek

**Affiliations:** 1 Center for Open Science, Charlottesville, Virginia, United States of America; 2 University of Belgrade, Belgrade, Serbia; 3 University of California, Riverside, Riverside, California, United States of America; 4 University College London, London, United Kingdom; 5 Max Planck Institute for Research on Collective Goods, Bonn, Germany; 6 Mississippi State University, Starkville, Mississippi, United States of America; 7 University of Vienna, Vienna, Austria; 8 University of Virginia, Charlottesville, Virginia, United States of America; University of Edinburgh, UNITED KINGDOM

## Abstract

Beginning January 2014, *Psychological Science* gave authors the opportunity to signal open data and materials if they qualified for badges that accompanied published articles. Before badges, less than 3% of *Psychological Science* articles reported open data. After badges, 23% reported open data, with an accelerating trend; 39% reported open data in the first half of 2015, an increase of more than an order of magnitude from baseline. There was no change over time in the low rates of data sharing among comparison journals. Moreover, reporting openness does not guarantee openness. When badges were earned, reportedly available data were more likely to be actually available, correct, usable, and complete than when badges were not earned. Open materials also increased to a weaker degree, and there was more variability among comparison journals. Badges are simple, effective signals to promote open practices and improve preservation of data and materials by using independent repositories.

## Abstract 2

Badges that acknowledge open practices significantly increase sharing of reported data and materials, as well as subsequent accessibility, correctness, usability, and completeness.

## Author Summary

Openness is a core value of scientific practice. The sharing of research materials and data facilitates critique, extension, and application within the scientific community, yet current norms provide few incentives for researchers to share evidence underlying scientific claims. In January 2014, the journal *Psychological Science* adopted such an incentive by offering “badges” to acknowledge and signal open practices in publications. In this study, we evaluated the effect that two types of badges—Open Data badges and Open Materials badges—have had on reported data and material sharing, as well as on the actual availability, correctness, usability, and completeness of those data and materials both in *Psychological Science* and in four comparison journals. We report an increase in reported data sharing of more than an order of magnitude from baseline in *Psychological Science*, as well as an increase in reported materials sharing, although to a weaker degree. Moreover, we show that reportedly available data and materials were more accessible, correct, usable, and complete when badges were earned. We demonstrate that badges are effective incentives that improve the openness, accessibility, and persistence of data and materials that underlie scientific research.

## Introduction

Transparency of methods and data is a core value of science [1–4] and is presumed to help increase the reproducibility of scientific evidence [5–6]. However, sharing of research materials, data, and supporting analytic code is the exception rather than the rule [7]. In fact, even when data sharing is required by journal policy or society ethical standards, data access requests are frequently unfulfilled [8–9], or available data are incomplete or unusable [10]. Moreover, data and materials become less accessible over time [11]. These difficulties exist in contrast to the value of openness in general and to the move toward promoting or requiring openness by federal agencies, funders, and other stakeholders in the outcomes of scientific research [12–14].

Why is there such a big gap between the value and practice of open data and materials? A simple answer is that there are pragmatic barriers to sharing and few incentives to overcome them [15]. The present academic culture emphasizes publications and grants as researchers’ primary incentives. Few journals or funders require public sharing of data or materials as a condition of publication or funding. More common are ineffective policies that encourage or require sharing upon request. The key problem with “upon request” policies is that requests come after the researcher has completed the research, published the article, and lost or forgot detailed materials and data [9,11,16]. Finding and reconstructing data or materials for others’ use after the fact is burdensome and fraught with error.

Less burdensome solutions involve preparing for sharing while actively working with the materials and data, rather than reconstructing that work in retrospect. However, even though researchers endorse openness and transparency [1], with many demands on their time, researchers are not likely to share data and materials unless they have incentives to do so. Furthermore, despite the endorsement of transparency, some publishers and funders are reluctant to require materials and data sharing or to enforce such requirements. In this article, we report evidence that a very simple incentive—badges acknowledging open practices—can dramatically increase sharing of data and materials. Moreover, simple specifications of what is required to earn a badge is effective in promoting use of independent repositories that have stronger assurance of availability and preservation than ad hoc methods such as posting data on personal websites [11].

### Badges as Signals of Open Practices

Signals rapidly communicate information such as values, beliefs, and identities to others [17–19]. Male peacocks signal fitness with elaborate feather displays, automobile drivers signal political identities with bumper stickers, and Chicagoans signal acceptance of yearly disappointment by wearing Cubs apparel.

Badges are an easy means of signaling and incentivizing desirable behaviors. Journals can offer badges acknowledging open practices to authors who are willing and able to meet criteria to earn the badge (https://osf.io/tvyxz/). Badges acknowledging open practices signal that the journal values transparency, lets authors signal that they have met transparency standards for their research, and provides an immediate signal of accessible data, materials, or preregistration to readers. Badges allow adopting journals to take a low-risk policy change toward increased transparency. Compared, for example, to measures that require data deposition as a condition of publication, badge implementation is relatively resource-lite, badges are an incremental change in journal policy, and if badges are not valued by authors, they are ignored and business continues as usual.

In January 2014, *Psychological Science* (*PSCI*) adopted badges to acknowledge open data, open materials, and preregistration of research if published. Following the specifications maintained by the Center for Open Science (http://cos.io/) Badges Committee for what it means to be “open data” or “open materials,” the *PSCI* editorial team awarded one or more badges to authors who applied for them upon article acceptance and provided evidence to the editors that they met the specified criteria. To meet the criteria to earn an open data badge, authors must make all digitally shareable data relevant to the publication available on an open access repository. Similarly, to earn an open materials badge, authors must make all digitally shareable materials, such as survey items, stimulus materials, and experiment programs, available on an open access repository. Materials that cannot be shared digitally must be described in sufficient detail for an informed reader to know how to reproduce the protocol.

Those who apply for a badge and meet open data or open materials specifications receive the corresponding badge symbol at the top of their paper and provide an explicit statement in the paper including a URL to the data or materials at an open repository. We did not include preregistration, the act of confirming an unalterable version of one’s research plan prior to collecting data, in this analysis. Preregistration requires initiating behaviors prior to starting the research and thus will require more time to see any impact in the published literature than in our assessment of impact within the first 1.5 years.

We examined the impact of adopting badges by comparing data and material sharing rates before (2012–2013; *PSCI* before badges) and after adoption (2014–May 2015; *PSCI* with badges) in *Psychological Science*, and across the same time period in comparison journals from the same discipline (journals without badges). Articles already in the publication process on January 1, 2014 may not have had an opportunity to apply for badges even though their article appeared in 2014. This suggests that the results reported in this article underestimate the overall impact of badges. The design and analysis of this study was preregistered at https://osf.io/ipkea/, and all data and materials are available at https://osf.io/rfgdw/. We preregistered an additional investigation involving reaching out to authors to evaluate accessibility of data or materials that were not shared upon publication. However, we postponed that part of data collection due to feasibility constraints.

## Method

### Sample

We used the population of empirical articles with studies based on experiment or observation (*N* = 2,478) published in 2012, 2013, 2014, and January through May 2015 issues of one journal that started awarding badges, *Psychological Science* (*PSCI*; *N* = 838), and four journals in the same discipline that did not: *Journal of Personality and Social Psychology* (*JPSP*; *N* = 419), *Journal of Experimental Psychology*: *Learning*, *Memory*, *and Cognition* (*JEPLMC*; *N* = 483), *Developmental Psychology* (*DP*; *N* = 634), and *Clinical Psychological Science* (*CPS*; *N* = 104). *Psychological Science* (2014 impact factor [IF] = 4.94) is a respected journal that publishes empirical research from any area of psychology. The four comparison journals (2014 IF’s 2.86 to 5.03) are respected journals that publish empirical research from a particular area of psychology represented by their titles. *Clinical Psychological Science*, which has only been publishing since January 2013, does not yet have an estimate of impact. It was selected to represent clinical psychology and as the other empirical journal published by the Association for Psychological Science. A total of 220 additional articles published in these journals between 2012 and May 2015 are not part of this corpus because they were not reports of empirical research (i.e., editorials, theoretical reviews, commentaries).

### Materials and Procedure

Coders were trained to reliably apply the coding scheme for assessing accessibility of data and research materials (https://osf.io/4rf3v/). Five trial articles from four journals included in this study, with content representative of a range of possible outcomes, were given to each coder. The first author’s coded responses to these articles were defined as the gold standard. Coders had to achieve 95% reliability with the gold standard before actual coding began. All questions, including free text answers, were included in this evaluation of reliability. If any response did not provide the same conclusion as the gold standard, it was marked incorrect. If 95% reliability was not met with the initial five, coders received three new trial articles and repeated the process. Once their responses were reliable, coders received additional coding instructions (https://osf.io/er9xk/) and access to the population of articles.

All articles had a unique, standardized identification number based on their order, month, year, and journal of publication. Coders selected articles solely by this identification number. Individual articles can be matched to their corresponding bibliographic metadata, available at https://osf.io/rmune/.

**Fig 1:**
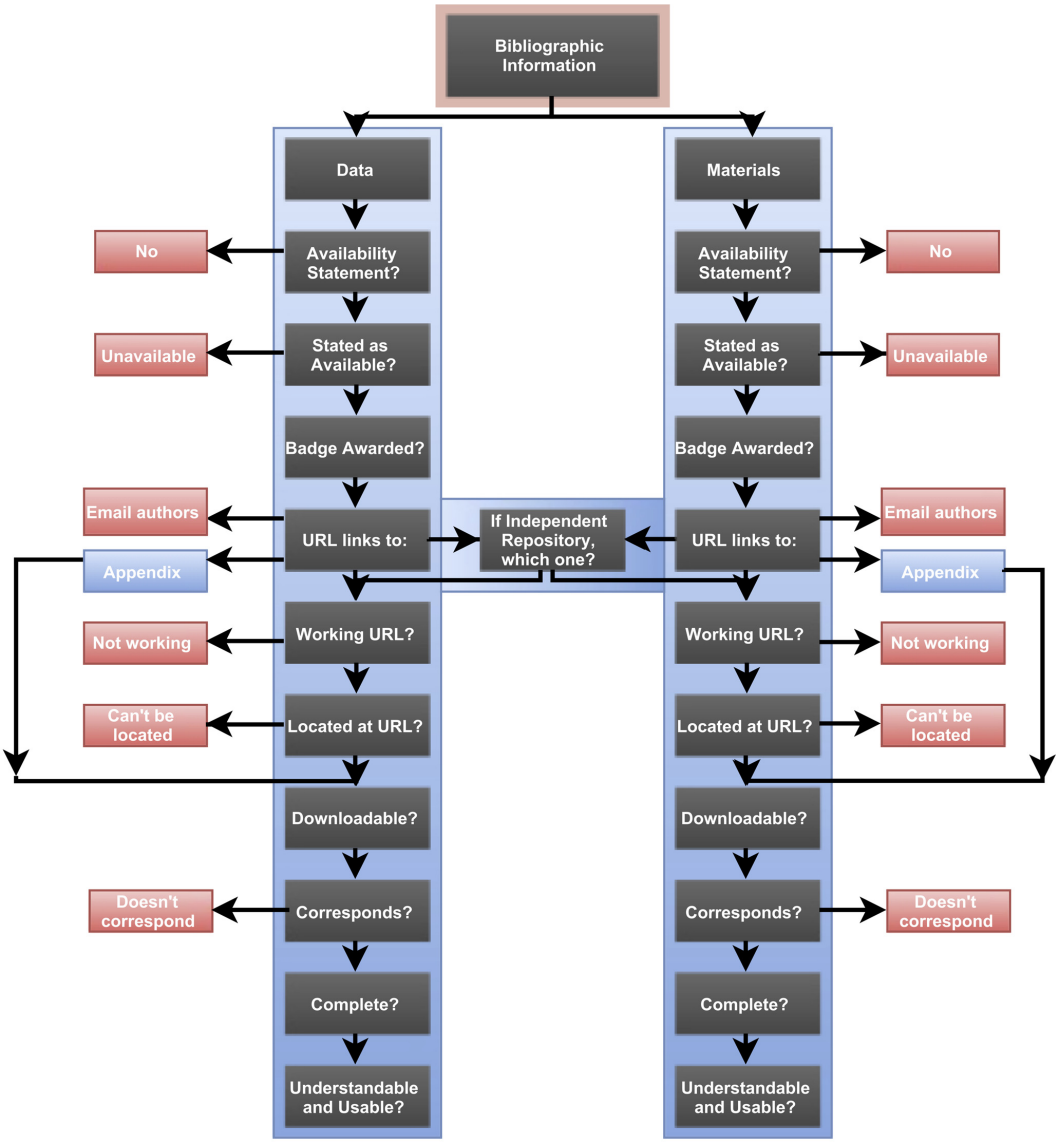
Article coding scheme. A visual illustration of the full coding scheme used to evaluate the availability of data and materials. This figure is available for download on the Open Science Framework at https://osf.io/kjsxv/.

Availability of data and materials were coded using an identical coding structure. The full coding scheme illustrated in Fig 1 is available at https://osf.io/4rf3v/. All of the variables used in analysis for this article are as follows:

**Badge awarded**. Whether or not the article was awarded a badge for open data or open materials.

**Availability statement**. Whether or not the article makes a statement regarding the location, available or unavailable, of its data or materials.

**Reported available**. Whether or not the article specifically states the data or materials were available for use, including noting that the data or materials are not available.

**Reported location**. If data or materials were available, what means were provided for accessing them: an independent archive/repository, personal website, independent website, journal supplement, appendix or table, or an indication that data or materials were available upon request.

**Actually available**. Whether the data or materials reported available at a publicly accessible location were found at the expected location, excluding articles with data or materials in the article text, appendix, or journal-hosted supplement.

**Correct data/materials**. If the data or materials could be retrieved, whether the data or materials corresponded to what was reported as being available. To determine correctness of open data, coders evaluated whether the type of data, the variables in the dataset, the number of participants, and the data contents matched the description provided in the manuscript. To determine correctness of open materials, coders evaluated whether the contents of the materials, such as survey items and stimuli, matched the description provided.

**Usable data/materials**. If data or materials could be retrieved, whether the data or materials were understandable and usable after brief review. To determine the usability of data/materials, coders evaluated whether they felt the format of and the context provided with the data/materials would allow them to easily be used for their own purposes.

**Complete data/materials**. If data or materials could be retrieved, whether all of the data or materials for reproducing the reported findings appeared to be available. To determine the completeness of the data/materials, coders evaluated whether all data/materials described for all studies in the article were accessible.

## Results

### Reported Availability of Data and Materials by Year and by Journal

We first examined whether articles’ reporting availability of data and materials increased over time, particularly in *Psychological Science* (*PSCI*) after badges were introduced on January 1, 2014. We examined the entire population of empirical articles from the target and comparison journals (*N* = 2,478); as such, we used descriptive statistics to evaluate our research questions.

**Fig 2:**
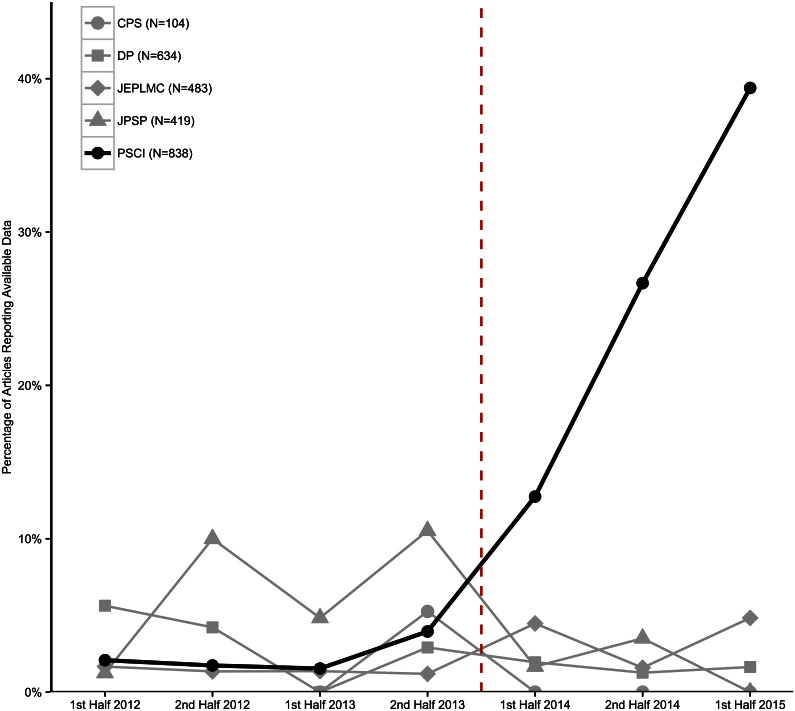
Reportedly available data. Percentage of articles reporting open data by half year by journal. Darker line indicates *Psychological Science*, and dotted red line indicates when badges were introduced in *Psychological Science* and none of the comparison journals. Underlying data (https://osf.io/a29bt/) and scripts (https://osf.io/bdtnq/) to reproduce this figure can be found on the Open Science Framework.

In *PSCI*, for the half years prior to 2014, an average of 2.5% of articles reported open data (range = 1.5% to 4.0% per half year), and after January 1, 2014, increased monotonically with an average of 22.8% of articles reporting open data (range = 12.8% to 39.4%; Fig 2). Across the four comparison journals, for the half years prior to 2014, an average of 3.3% of articles reported open data (range = 1.6% to 4.9%), and after January 1, 2014, the average was 2.1% (range = 1.8% to 2.3%). All four comparison journals had very low rates of articles reporting data availability (*JPSP* = 4.5%, *JEPLMC* = 2.3%, *DP* = 2.4%, *CPS* = 1.0%).

**Fig 3:**
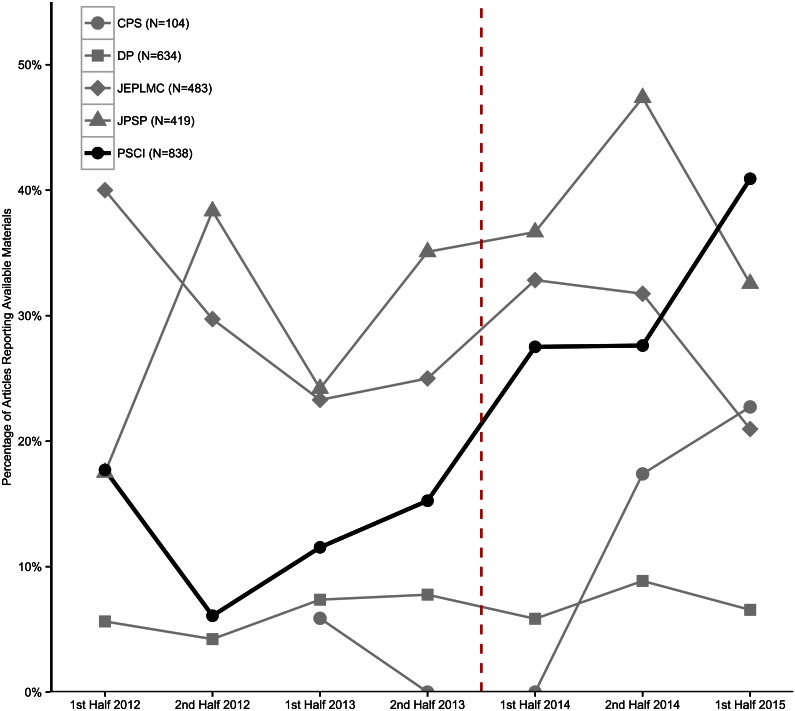
Reportedly available materials. Percentage of articles reporting open materials by half year by journal. Darker line indicates *Psychological Science*, and dotted red line indicates when badges were introduced in *Psychological Science* and none of the comparison journals. Underlying data (https://osf.io/a29bt/) and scripts (https://osf.io/bdtnq/) to reproduce this figure are available on the Open Science Framework.

In *PSCI*, for the half years prior to 2014, an average of 12.7% of articles reported open materials (range = 6.1% to 17.7%), and after January 1, 2014, increased monotonically with an average of 30.3% reporting open materials (range = 27.5% to 41.0%; Fig 3). Across the four comparison journals, for the half years prior to 2014, an average of 19.3% of articles reported open materials (range = 16.2% to 23.4%), and after January 1, 2014, 20.6% (range = 17.4% to 26.1%). The four comparison journals varied widely in rates of reporting materials availability (*JPSP* = 32.2%, *JEPLMC* = 28.8%, *DP* = 6.6%, *CPS* = 9.6%). The social- and cognitive-psychology-oriented journals tended to report sharing materials more frequently. Usually, those were descriptions of surveys or stimulus items in an appendix for the article, rather than a complete description of the protocol.

In summary, reported sharing of materials and especially data increased dramatically in *Psychological Science* after introducing badges, but did not change systematically in the comparison journals over the same time period.

### Reported Location of Available Data and Materials

We next examined whether the introduction of badges was associated with an increase in the rate of using independent repositories. Repositories may provide greater quality assurance and guarantees of preservation than other storage locations such as personal web pages. For this analysis, we considered only those articles that reported sharing.

Among *PSCI* articles reporting available data, 7.7% (*N* = 1) before January 1, 2014, and 71.2% (*N* = 52) after, reported that the data were available in an independent repository. Among comparison journal articles reporting available data, 9.7% (*N* = 3) before January 1, 2014, and 26.7% (*N* = 4) after, reported that the data were available in an independent repository. This suggests that, when data is shared, it is increasingly likely over time to be shared in an independent repository, and that availability of badges dramatically accelerates this trend. In fact, 46 of the 73 *PSCI* articles reporting data availability in 2014 and 2015 also earned a badge, and 100% of those 46 reported being in an independent repository.

Similarly, among *PSCI* articles reporting available materials, 0% (*N* = 0) before January 1, 2014, and 45.4% (*N* = 44) after reported that the materials were available in an independent repository. Among comparison journal articles reporting available materials, 0% *(N* = 0) of materials before January 1, 2014, and 2.0% (*N* = 3) after reported that the materials were available in an independent repository. Again, 38 of the 97 *PSCI* articles reporting materials availability in 2014 and 2015 also earned a badge, and 100% of those 38 reported being in an independent repository.

### Actual Availability, Correctness, Usability, and Completeness of Reported Open Data and Materials

The first results showed nearly a 10-fold increase in reported availability of data for *PSCI* with badges (22.8%), compared with *PSCI* before badges (2.5%) and the four journals without badges combined (2.8%). Effects were similar but weaker for materials (30.3%, 12.7%, and 19.9%, respectively). However, reporting availability of data and materials does not guarantee that they are available, or that they are correct, usable, and complete.

Do badges increase the likelihood that reported available data and materials are actually available, correct, usable, and complete? It is possible that badging is sufficient to increase motivation to claim the behavior, but not sufficient to increase performing the behavior. However, the specified criteria for earning the badge, the simple editorial checks on meeting those criteria, and the visibility of the badge may all stimulate sharing behavior.

#### Actual data sharing

**Fig 4:**
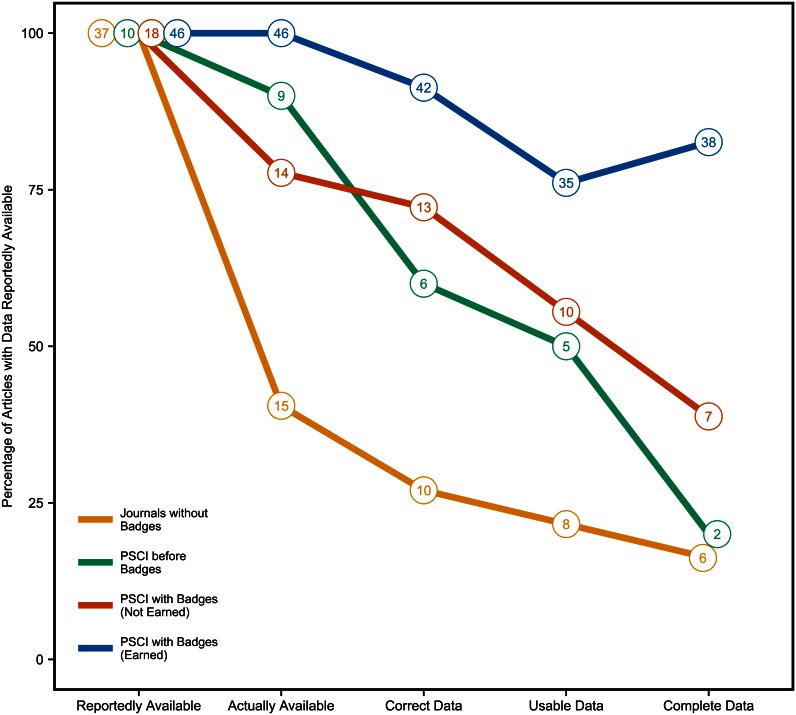
Actually available, correct, usable, and complete data. Percentage of articles with data reported available at an independent archive or personal website that were actually available, had correct data, had usable data, and had complete data. Once *Psychological Science* started offering badges, some articles reported availability but either did not apply for or earn a badge; others reported availability and did earn a badge. These are represented separately. Total number of articles reported in data points. Underlying data (https://osf.io/srgjb/) and scripts (https://osf.io/d78cf/) to reproduce this figure are available on the Open Science Framework.

For this analysis, we restricted the dataset to only articles reporting that data and materials were available in an independent archive or personal website. Results of the proportion of articles that were available, correct, usable, and complete compared to the number of articles claiming data availability are presented in Fig 4. “Correct data” means that coders agreed that the data corresponded to the description of the data reported available in the paper. “Usable data” means data were understandable and estimated to be usable upon brief review. “Complete data” means the data for all reported studies were available and perceived by coders to be sufficient to reproduce the reported results (coders did not actively reanalyze the data).

Journals without badges had 2.8% of articles reporting data availability (*N* = 46): nine (19.6%) in the article, supplement, or by request, and 37 (80.4%) at an independent repository, personal website, or independent website. Of just those reportedly available at a website or repository, just 40.5% (*N* = 15) were actually available, 27.0% (*N* = 10) had correct data, 21.6% (*N* = 8) had usable data, and 16.2% (*N* = 6) had complete data. *PSCI* before badges had 2.5% of articles reporting data availability (*N* = 13): three in the article, supplement, or by request, and 10 at an independent repository, personal website, or independent website. Of just those reported at a website or repository, 90.0% (*N* = 9) were actually available, 60.0% (*N* = 6) had correct data, 50.0% (*N* = 5) had usable data, and just 20.0% (*N* = 2) had complete data.

*PSCI* with badges had 22.8% of articles reporting data availability (*N* = 73): nine (12.3%) in the article, supplement, or by request, and 64 (87.7%) at an independent repository, personal website, or independent website. Of just those reported at a website or repository, 93.8% (*N* = 60) were actually available, 85.9% (*N* = 55) had correct data, 70.3% (*N* = 45) had usable data, and 70.3% (*N* = 45) had complete data. When badges were available, rates of actual availability, correctness, usability, and completeness were dramatically higher than the comparison journals, but not close to perfect. However, not all of the *PSCI* with badges articles reporting availability actually earned a badge. When we split the data between those reporting data availability with a badge (*N* = 46) and those reporting data availability without a badge (*N* = 18), we observed that among badge earners, 100% (*N* = 46) were actually available, 91.3% (*N* = 42) were correct data, 76.1% (*N* = 35) were usable data, and 82.6% (*N* = 38) were complete data. However, among those without a badge, 77.7% (*N* = 14) were actually available, 72.2% (*N* = 13) were correct data, 55.6% (*N* = 10) were usable data, and 38.9% (*N* = 7) were complete data.

#### Actual materials sharing

**Fig 5:**
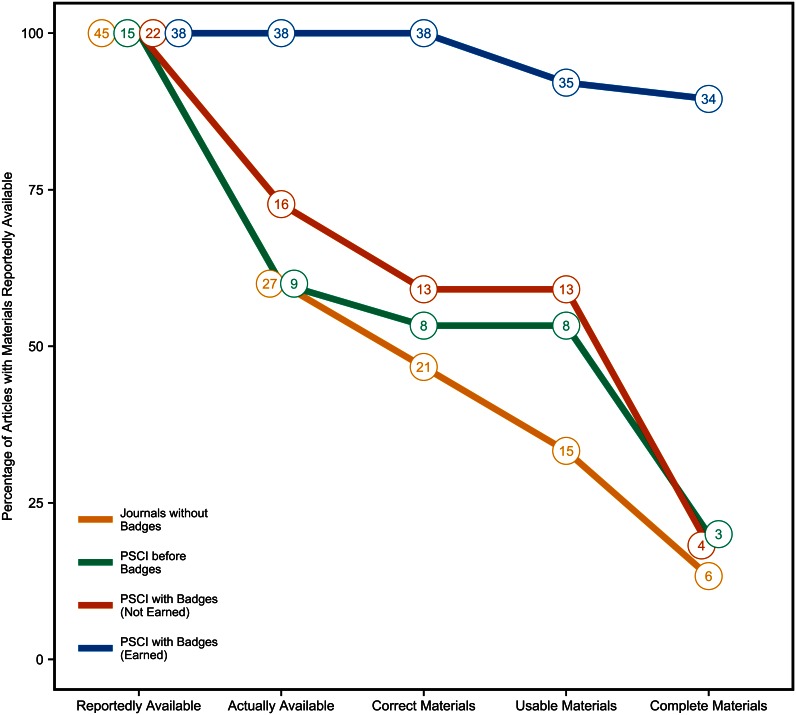
Actually available, correct, usable, and complete materials. Percentage of articles with materials reported available at an independent archive or personal website that were actually available, had correct materials, had usable materials, and had complete materials. Once *Psychological Science* started offering badges, some articles reported availability but did not earn a badge, and others reported availability and did earn a badge. These are represented separately. Total number of articles reported in data points. Underlying data (https://osf.io/8ds2g/) and scripts (https://osf.io/f7kqr/) to reproduce this figure are available on the Open Science Framework.

We did the same analysis for materials availability (Fig 5). An important difference between sharing data and materials is that it was more common that at least some of the materials were shared in the article itself.

Journals without badges had 19.9% of articles reporting materials availability (*N* = 326), 281 (86.2%) in the article, supplement, or by request and 45 (13.8%) at an independent repository, personal website, or independent website. Of just those reportedly available at a website or repository, 60.0% (*N* = 27) were actually available, 46.7% (*N* = 21) were correct materials, 33.3% (*N* = 15) were usable materials, and 13.3% (*N* = 6) were complete materials.

*PSCI* before badges had 12.7% of articles reporting materials availability (*N* = 66), 51 (77.3%) in the article, supplement, or by request and 15 (22.7%) at an independent repository, personal website, or independent website. Of those reportedly available at a website or repository, 60.0% (*N* = 9) were actually available, 53.3% (*N* = 8) were correct materials, 53.3% (*N* = 8) were usable materials, and 20.0% (*N* = 3) were complete materials.

*PSCI* with badges had 30.3% of articles reporting materials availability (*N* = 97), 37 (38.1%) in the article, supplement, or by request and 60 (61.9%) at an independent repository, personal website, or independent website. Of those reportedly available at a website or repository, 90.0% (*N* = 54) were actually available, 85.0% (*N* = 51) were correct materials, 80.0% (*N* = 48) were usable materials, and 63.3% (*N* = 38) were complete materials. As with data, badges increased the rate of actual, correct, usable, and complete sharing of materials compared to journals without badges, but was not close to perfect. Again, not all of the *PSCI* with badges articles reporting availability earned a badge. When we split the data between those reporting materials availability with a badge (*N* = 38) and those reporting materials availability without a badge (*N* = 22), we observed that among badge earners, 100% (*N* = 38) were actually available, 100% (*N* = 38) were correct materials, 92.1% (*N* = 35) were usable materials, and 89.5% (*N* = 34) were complete materials. However, among those that reported availability without a badge, 72.7% (*N* = 16) were actually available, 59.1% (*N* = 13) were correct materials, 59.1% (*N* = 13) were usable materials, and 18.2% (*N* = 4) were complete materials.

These dramatic differences between articles with and without badges supports the interpretation that the badges, particularly the criteria for earning them, strongly influenced actual availability, correctness, usability, and completeness.

## Discussion

In the present scientific culture, there are few direct positive incentives that encourage researchers to make data and materials publicly accessible. The result is that a tiny minority of data from published articles are shared [7,20]. Across four prominent psychology journals, plus *Psychological Science* prior to adopting badges, less than 3% of articles reported having publicly accessible data. By contrast, after *Psychological Science* adopted badges, reported sharing rates increased about 10-fold overall and to nearly 40% in the last half year examined (first half of 2015).

Notably, without badges, the paltry percentage of reported sharing is a gross exaggeration of actual sharing. Less than 1% of articles had publicly accessible data that was usable or complete. In fact, just six of 37 articles from journals without badges and two of 10 articles from *PSCI* before badges that reported available data had accessible, correct, usable, complete data. On the other hand, with badges, actual sharing was very similar to reported sharing. In sum, badges were associated with a dramatic increase in reported sharing and, on top of that, a dramatic increase in actual data sharing.

Effects on sharing research materials were similar but weaker, with badges producing “only” about three times more sharing. The weaker effect compared to data is likely due to the higher overall rates of sharing at baseline. For example, the social and cognitive psychology comparison journals showed quite high sharing rates—most of that being attributable to sharing survey items or other stimulus materials in the article text, tables, or appendices. Notably, while our coding scheme counted this as sharing materials, many fewer of these cases were complete sharing of all materials. As such, badges may facilitate the more complete sharing of materials, even in domains for which sharing some materials is already relatively common.

### Limitations

The present investigation leverages a naturalistic intervention that occurred at *Psychological Science* and not at similar journals in the same discipline. However, opportunities to earn badges were not randomly assigned to journals or authors. This necessarily weakens the certainty of causal inference. Nonetheless, we assert a causal interpretation that badges promote data sharing because of the implausibility of alternative explanations.

The most obvious alternative explanation is that the adoption of badges changed the population of authors submitting or earning acceptance at *Psychological Science* dramatically toward that very small minority (<3%) that shares data and materials even when badges are not offered. Given that *Psychological Science* has extremely high rejection rates (~93%), such a scenario would require a rapid and sizable shift in population submitting to the journal [21]. In comparison, rejection rates at *Clinical Psychological Science*, *Developmental Psychology*, *Journal of Experimental Psychology*: *Learning*, *Memory*, *and Cognition*, and *Journal of Personality and Social Psychology* are ~77%, 80%, 78%, and 89%, respectively (personal communication, [22]). Also, given *Psychological Science*’s status, concerns about optional sharing would need to exceed the perceived value of publishing in the field’s premiere empirical outlet. Moreover, many of the manuscript submissions of articles published in 2014 would have occurred in 2013—prior to the announcement of the new policy.

Another weakness in the present research is that the evaluations of data and material accessibility, correctness, usability, and completeness were the result of coder assessments and did not include reanalysis of the data or reuse of the materials. Such an effort would provide complementary insight on the extent to which this increase in transparency translates to an increase in reproducibility. Another research opportunity with the existing data is to code the research domains of the *PSCI* articles with badges to see if data and materials sharing rates accelerated more quickly in some subfields compared to others. All data reported in this paper are available at https://osf.io/u6g7t/ to facilitate follow-up investigation.

Finally, badges are not a panacea. Sharing rates increased dramatically, but not all data or materials that could be shared were shared. Moreover, even with badges, the accessibility, correctness, usability, and completeness of the shared data and materials was not 100%. Some incompleteness could be attributable to gaps in the specifications for earning badges. For example, in late 2015, the Center for Open Science Badges Committee (http://osf.io/tvyxz) considered provisions for situations in which the data or materials for which a badge was issued somehow disappear from public view. Adherence to badge specifications can also be improved by providing easy procedures for editors or journal staff to validate data and material availability before issuing a badge, and by providing community guidelines for validation and enforcement.

### Next Steps

Broader adoption of badges across journals will accelerate the accumulation of evidence about their effectiveness and will facilitate the refinement of specifications for badge awards and the process of badge administration. For example, the Center for Open Science is collaborating with publishers and others to create a badge “bakery” that inserts metadata about the issuer, recipient, and location of resources into the badge itself. As digital objects, the badges could then be searched, indexed, and maintained programmatically, which would increase their value for monitoring transparent practices.

Badges will not be sufficient to make transparent all data and materials that could be made publicly accessible. Additional interventions will include government, funder, or publisher mandates, such as some of the more stringent standards offered in the Transparency and Openness Promotion (TOP) Guidelines (http://cos.io/top/) [14]. Evaluation of different mechanisms for promoting or requiring transparency will help reveal the most efficient and effective methods. And, of course, 100% availability is unlikely because of ethics, privacy, and intellectual property exceptions. However, badges and additional interventions can shift the culture to make sharing the default.

## Conclusion

Badges may seem more appropriate for scouts than scientists, and some have suggested that badges are not needed [23]. However, actual evidence suggests that this very simple intervention is sufficient to overcome some barriers to sharing data and materials. Badges signal a valued behavior, and the specifications for earning the badges offer simple guides for enacting that behavior. Moreover, the mere fact that the journal engages authors with the possibility of promoting transparency by earning a badge may spur authors to act on their scientific values. Whatever the mechanism, the present results suggest that offering badges can increase sharing by up to an order of magnitude or more. With high return coupled with comparatively little cost, risk, or bureaucratic requirements, what’s not to like?

## Abbreviations

*CPS*: *Clinical Psychological Science*
*DP*: *Developmental Psychology*
*JEPLMC*: *Journal of Experimental Psychology*: *Learning*, *Memory*, and *Cognition*
*JPSP*: *Journal of Personality and Social Psychology*
*PSCI*: *Psychological Science*
IF: Impact Factor
